# Clinical characteristics, medication use, and impact of primary headache on daily activities: an observational study using linked online survey and medical claims data in Japan

**DOI:** 10.1186/s12883-023-03122-9

**Published:** 2023-02-21

**Authors:** Koichi Hirata, Hiromi Sano, Hiroyuki Kondo, Yoshiyuki Shibasaki, Nobuyuki Koga

**Affiliations:** 1 Dokkyo Medical University, 880 Kitakobayashi, Mibu, Shimotsuga, Tochigi, Japan; 2 Medical Affairs, Otsuka Pharmaceutical Co., Ltd., 3-2-27 Otedori, Chuo-Ku, Osaka, Japan; 3 Medical Affairs, Otsuka Pharmaceutical Co., Ltd., 2-6-14 Konan, Minato-Ku, Tokyo, Japan; 4 Medical Affairs, Otsuka Pharmaceutical Co., Ltd., 463-10 Kagasuno, Kawauchi-Cho, Tokushima, Japan

**Keywords:** Prevalence, Migraine, Tension-type headache, Cluster headache, Quality of life, Ms-QOL, WPAI

## Abstract

**Background:**

Limited epidemiological data are available for headache disorders in Japan, and no recent studies have reported the impact of several primary headache disorders in Japan. This study aimed to report the up-to-date epidemiological data and impact of primary headaches on daily activities as well as the use of medical care, clinical features, and pain severity/activity impairment using nationwide data in Japan.

**Methods:**

We used anonymized online survey data coupled with medical claims data, from individuals aged 19–74 years old, that were provided by DeSC Healthcare Inc. The outcomes included the prevalence of migraine, tension-type headache, cluster headache, and other headache types stratified by age and sex, use of medical care, clinical features, medication use, and severity of pain/activity impairment. All outcomes were examined separately for each headache type. This is the second paper reported concurrently with this research.

**Results:**

The study population comprised 691/1,441/21/5,208 individuals with migraine/tension-type headache/cluster headache/other headache types, respectively. The prevalence of migraine and tension-type headache was higher in women than in men but was similar for cluster headache (male vs. female, 1.7% vs. 7.4%, 5.3% vs. 10.8%, and 0.1% vs. 0.1%, respectively). The percentage of individuals with migraine, tension-type headache, cluster headache who had not seen a doctor was 81.0%, 92.0%, 57.1%, respectively. The common headache triggers were fatigue in migraine and tension-type headache, and weather-related phenomena and turning of the seasons in migraine. Common activities refrained from or reduced by headaches were “operating a computer or smartphone”, “drinking alcohol”, and “going to crowded places” in all three headache types and housework-related activities in women. Among individuals taking medicines, 16.8%, 15.8%, 47.6% with migraine, tension-type headache, and cluster headache reported moderate to severe pain, respectively, and 12.6%, 7.7%, 19.0% reported moderate to severe disability, respectively.

**Conclusions:**

This study found various triggers of headache attacks, and daily activities refrained from or reduced by headaches. Additionally, this study suggested that the disease burden in people possibly experiencing tension-type headaches, many of whom had not seen a doctor. The study findings are of clinical value for the diagnosis and treatment of primary headaches.

**Supplementary Information:**

The online version contains supplementary material available at 10.1186/s12883-023-03122-9.

## Background

Headache is globally the most prevalent neurological disorder [1] and is the fifth leading health problem in women in Japan [2]. According to the International Classification of Headache Disorders, 3rd edition (ICHD-3), headache disorders are classified into secondary headaches with underlying causes, and primary headaches without such causes [3]. Primary headaches are further classified into several types such as migraine, tension-type headache, and trigeminal autonomic cephalalgias including cluster headache [3]. Although it is considered that primary headaches do not cause any organic damage to the brain, headache attacks greatly interfere with various aspects of daily activities. It is therefore desirable to improve daily activities and quality of life of those affected through diagnosis and treatment [4].

Limited epidemiological data are available for headache disorders in Japan, to date, seven epidemiological reports have been published in adults [5–11]. These reports documented the burden of migraine, and tension-type headache [6], on daily activities. However, such information is still largely missing for tension-type headache and cluster headache, and no epidemiological data for cluster headache have been reported in Japan. Sakai and Igarashi [5] reported that 74.0% of individuals with migraine complained of moderate or severe disability when conducting daily activities. Despite these findings, approximately 69.4% of individuals never consulted a doctor [5]. Takeshima et al. [6] also reported that similarly large proportions of people with migraine never consulted a doctor (61.0% and 71.8% in those with and without aura, respectively). Additionally, recent studies have shown the deleterious impact of migraine on quality of life and work performance [7, 9–11].

To date, only two epidemiological studies have been published on primary headache disorder of migraine diagnosed according to ICHD-3 [9, 11]. Although it is important that headache disorders are properly diagnosed and treated to improve daily activities of those affected [4], some individuals have overlapping symptoms (e.g., migraine and tension-type headache) [12], making classification difficult. With this in mind, the aim of this study was to report the up-to-date epidemiological data and impact of primary headaches classified according to the ICHD-3 on daily activities as well as use of the medical care, clinical features, and pain severity and activity impairment using a nationwide online survey using questionnaires coupled with medical claims. We focused on primary migraine, tension-type headache, cluster headache, and other headache types. To the best of our knowledge, our study is one of the first to concurrently report epidemiological data for several headache types globally and the first to report such data for cluster headache in Japan. This is the second paper of the research by Sakai et al. [13] that reports the prevalence of migraine and the treatment status in Japan. We report prevalence of each headache type stratified by age and sex, and the overall prevalence is reported in the paper by Sakai et al. [13].

## Methods

### Study design and data source

This study used the medical claims and questionnaire data from online surveys provided by DeSC Healthcare, Inc. (Tokyo, Japan; hereafter, DeSC). The data comprised anonymously processed information provided by the Society-Managed Employment-Based Health Insurance to DeSC prior to the start of this study. Therefore, this study used only anonymously processed information that had already been created. The study subjects included employees (approximately 550,000 people) aged 19–74 who work for large companies on a nationwide scale, including subscribers (approximately 200,000 people) who registered for the mHealth web service “kencom^®^” provided by DeSC. The online questionnaire contained 69 closed-ended questions, with single and multiple answers. Detailed information on the claims data and online survey are described in Sakai et al. [13].

### Ethics statement

As this survey used only anonymized data and the authors, data analysts, and any parties involved in the study including Otsuka Pharmaceutical Co., Ltd. and Clinical Study Support Inc. did not possess or receive correspondence sheets, it was impossible for those involved to identify individuals included in the study. As the study using anonymized data is outside the scope of the national guidelines “Ethical Guidelines for Medical and Health Research Involving Human Subjects”, the ethical committee review and individual-level consent were not required. However, the study protocol was approved by the ethics committee of the Research Institute of Healthcare Data Science (approval No.: RI2020012). Additionally, the survey was conducted in consideration of the Declaration of Helsinki (revised October 2013) by the World Medical Association and the Ethical Guidelines for Medical Research Involving Human Subjects.

### Study population

We extracted questionnaire response data collected from online surveys and medical claims receipt data from the database for the past 3 years including the month in which the survey was conducted from 1 December 2017 to 30 November 2020. The study population included all individuals whose response data were available.

### Outcome measures

The outcome included the prevalence of migraine, tension-type headache, cluster headache, and other headache types, stratified by age (19–29, 30–39, 40–49, 50–59, and 60–74 years old) and sex (male or female)*.* The headache type and its probable cases were classified based on the questionnaire responses including internal diagnostic criteria according to ICHD-3 [3]; Additional file 1). Individuals not classified in migraine, tension-type headache, and cluster headache were included in other headache types. The questions for the classification asked about headache in the past 30 days.

The use of medical care, based on questionnaire response, was categorized as following: frequency of current medical visits (regularly visited, not regularly visited, and not visited) in the past 6 months; frequency of medical visits in the past 6 months before answering the survey (≥ once/week, once/2 weeks, once/month, once/2 months, once/3 months, < once/3 months); reasons for initially seeing a doctor for headache (top four responses: unable to tolerate headaches, worried about other brain diseases, increased headache frequency, and over-the-counter [OTC] analgesics no longer effective); reasons for seeing a doctor once for headache and not seeing thereafter (top four responses: relieved not to have brain disease that threatened life, too much trouble, no time, and prescription drugs ineffective); and reasons for not seeing a doctor in the past 3 years (top four responses: OTC analgesics effective, used to having a headache, spontaneously resolving after endurance, and pain not sufficiently severe). All responses were provided for the first two questions whereas the top four responses in the migraine group were provided for the last three questions for all headache types, and all responses were categorical.

Clinical features and symptoms, based on questionnaire response, were classified as following: symptoms associated with headache (top nine responses: nausea or vomiting, stiff shoulder, neck pain, photophobia, phonophobia, dizziness, osmophobia, weakness or lethargy, and teary eye on the side of headache); site of pain (unilateral, bilateral, frontal, occipital, periorbital, and other); time of day of headache onset (upon waking, morning, afternoon, evening, other, and no particular time); headache triggers (top 18 responses: fatigue, stress, bad weather such as the time of typhoon, lack of sleep, turning points of the seasons, sunny or rainy days, work or housework, menstruation, excessive sleep, feeling nervous, weekdays, weekends [including holidays], drinking alcohol, release from nervousness, no particular triggers, smell of perfume or cigarettes, and sleep); activities that were interfered by headache (top seven responses: no focus on work or study, unable or unwilling to conduct housework, unwilling to work or study, cancelling plans or appointments, absence from work or school, unable to go outside, and unable to stay in crowded places); and activities that were refrained from or reduced by headache (operating a computer or smartphone, drinking alcohol, going to crowded places, exercising such as playing sports and walking, driving a car, housework [excluding grocery shopping, laundry, and cooking], socializing with friends and playing with children, going to grocery shopping, taking public transportation, cooking, taking a bath, doing laundry, dropping-off and picking-up children or family members, socializing with neighbors, putting on make-up, and other). Time of day of headache onset was stratified by the aforementioned age categories, and activities refrained from or reduced by headache stratified by sex.

Medication use, based on questionnaire responses and medical claims data, was classified as following: medication use in the past 6 months (no prescription drugs, OTC analgesics only, prescription drugs only [acute and prophylactic], and OTC and prescription drugs); number of OTC analgesics types (1 or ≥ 2); and types of prescription drugs for prophylactic treatments (antidepressants, anti-epileptics, calcium channel blockers, angiotensin-receptor blockers/angiotensin converting enzyme inhibitors, beta-blockers, and other) and for acute treatments (acetaminophen, non-steroidal anti-inflammatory drugs [NSAIDs], triptans, ergotamine antiemetic drugs, and other) in the past 6 months.

Severity of pain and activity impairment, based on survey response, was classified as following: severity of pain when not taking or taking medicines (severe = extreme pain or quite a bit of pain, moderate = moderate pain, mild = little pain or no pain); impairment in daily activities (severe = extreme difficulty or severe disruption in daily life, moderate = moderate difficulty in daily life, mild = slightly interferes with daily life or no trouble at all); hoped reduction in the number of headache in a month for improving daily life (slight, almost half, almost none, reduction in pain intensity per attack rather than reduction in pain frequency); migraine-specific quality of life (MS-QOL) scores [14]; and Work Productivity Activity Impairment (WPAI) scores [15]. MS-QOL was estimated using MSQ version 2.1, which is a 14-item questionnaire measuring the impact of migraine during the past 4 weeks across three domains: role function-restrictive that measures functional limitations on daily, work, and social activities (seven items); role function-preventive that measures functional prevention on daily, work, and social activities (four items); and emotional function that measures the impact on emotion (three items) [16, 17]. The source response data were scaled to range from 0 to 100, with higher score indicating better quality of life [16]. WPAI scores were estimated using the WPAI Questionnaire-General Health for the last 7 days before responding to the survey as follows: 1) percentage of work time missed in the last week due to health conditions (absenteeism); 2) percentage of impairment while working due to health conditions (presenteeism); 3) percentage of overall work impairment due to health conditions; and 4) percentage of activity impairment due to health conditions [15].

### Statistical analysis

All variables were descriptively summarized, with mean (standard deviation [SD]) and median (minimum, maximum) for continuous variables, and number and percentage for categorical variables, for each type of headache. The chi-square test was used to compare percentages between sexes for each response of activities refrained from or reduced by headaches. All statistical analyses were performed in SAS Release 9.4 (SAS Institute, Inc., NC, USA).

## Results

**Fig. 1:**
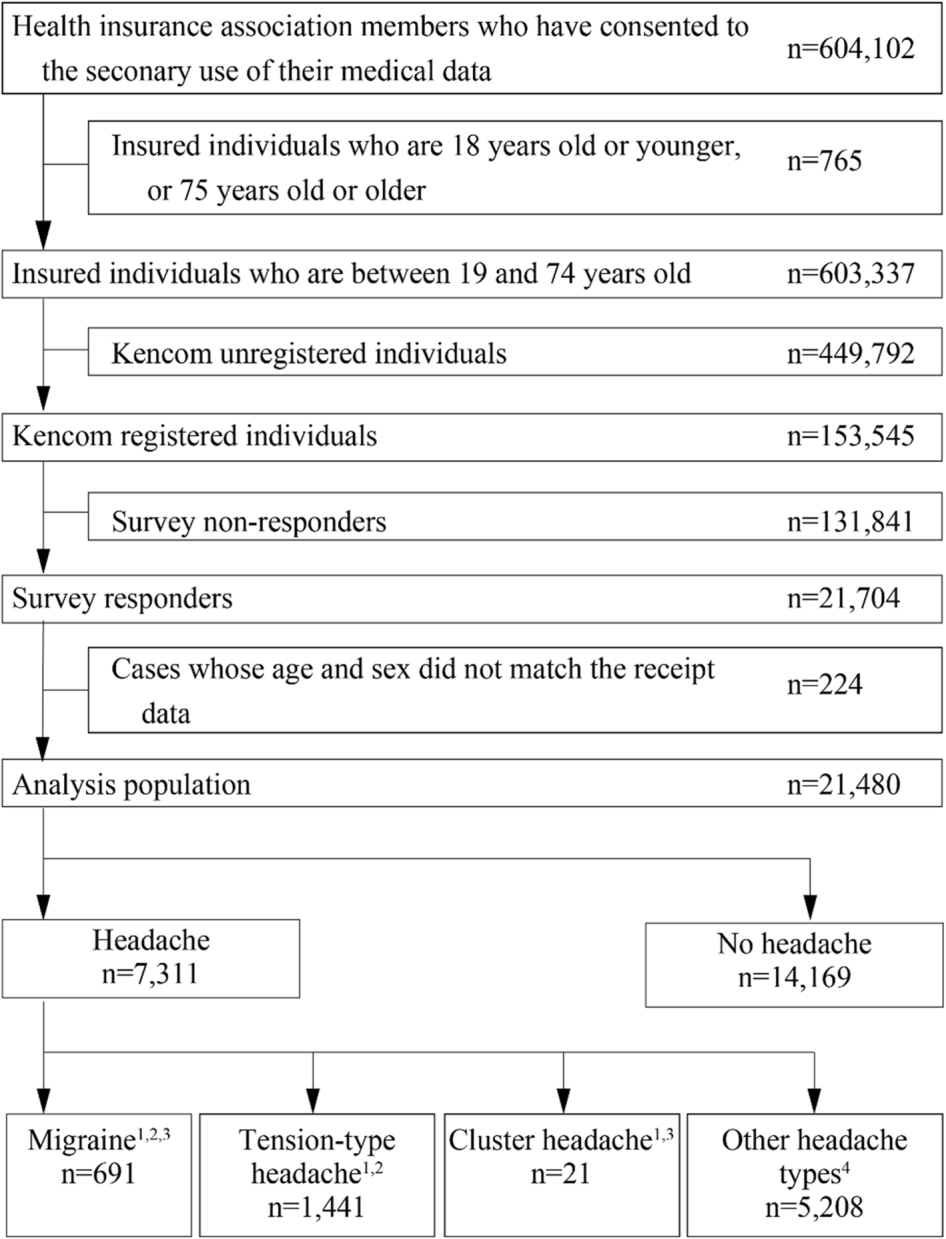
Patient disposition. Notes: ^1^ Each group except the group of other headache types included individuals classified as probable migraine, probable tension-type headache, or probable cluster headache, respectively. ^2^ There were 42 individuals who were classified into both migraine and tension-type headache. ^3^ There were 8 individuals who were classified into both migraine and cluster headache. ^4^ Post-hoc analysis of the “other headache types” by Sakai et al. [13] showed 261 people who had two or more matches with ID Migraine [18] and 286 people who had two or more matches with the 4-item simple migraine screener [19], both of which were modified versions for Japanese

Of the 604,102 individuals identified in the DeSC database, 7,311 and 14,169 individuals self-reported having or not having headaches, respectively (Fig. 1). Of those with headaches, 691/1,441/21/5,208 individuals were classified as migraine/tension-type headache/cluster headache/other headache types, respectively. Each headache type included individuals classified as probable, and 42 and 8 individuals were classified into both migraine and tension-type headache or cluster headache, respectively.

### Prevalence stratified by age and sex

**Fig. 2:**
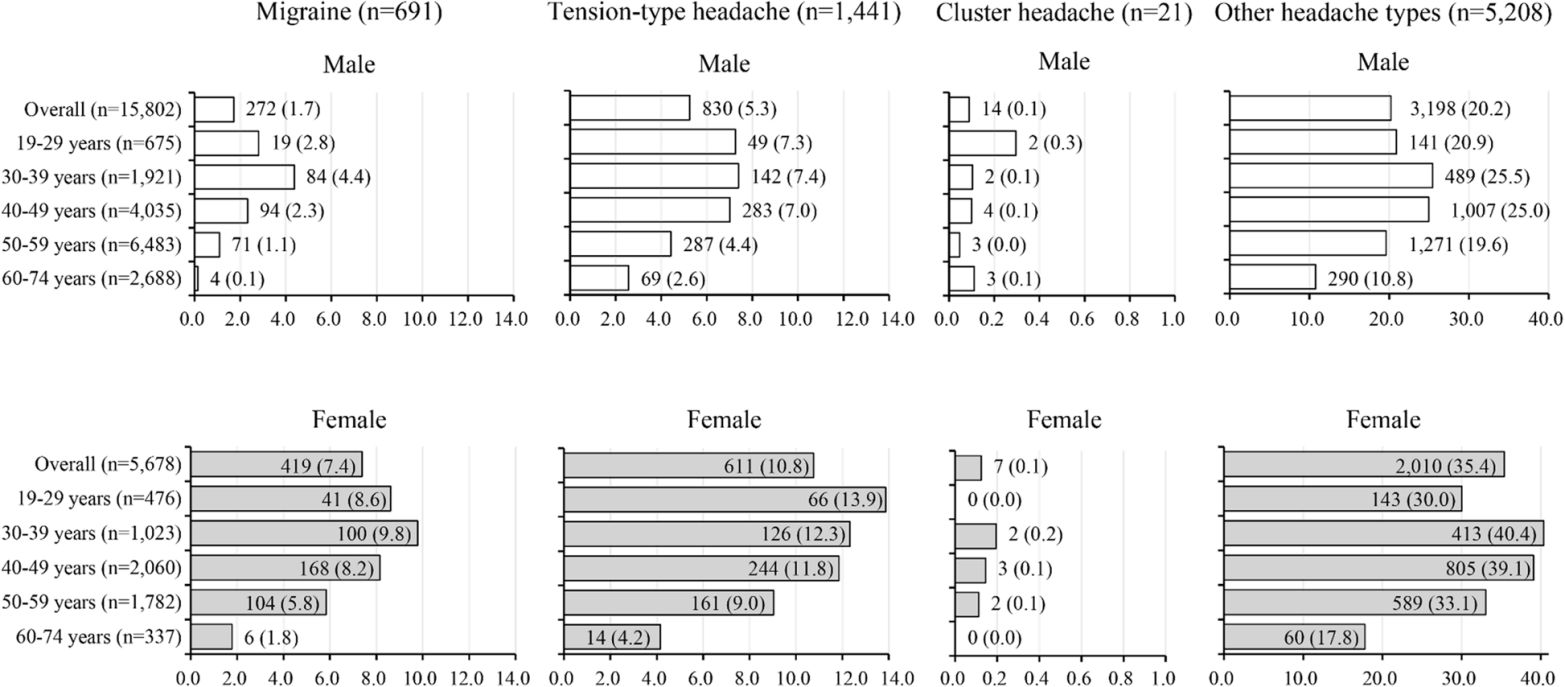
Prevalence of headache disorders. Notes: Data are expressed as n (%)

The prevalence of migraine and tension-type headache was higher in women than in men but was similar in cluster headache (male vs. female, 1.7% vs. 7.4%, 5.3% vs. 10.8%, and 0.1% vs. 0.1%; Fig. 2). The prevalence of migraine and tension-type headache was higher in ages 19–29 and 30–39 years, followed by 40–49 years in both men and women. Although the number of cases was small, the prevalence of cluster headache was higher in men aged 19–29 years.

**Fig. 3:**
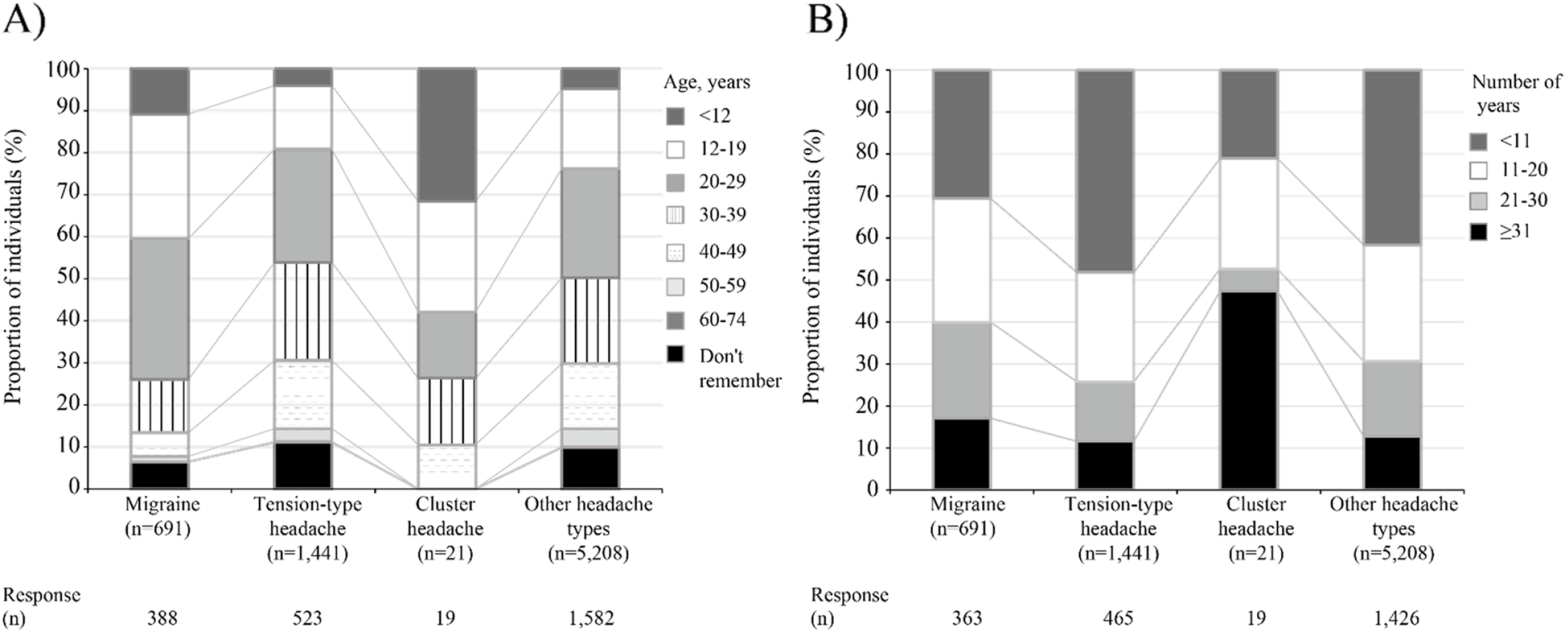
Age of headache onset (**A**) and years lived with headache (**B**). Notes: Response (n) represents the number of individuals with responses. Age of headache onset and years lived with headache were categorical, and the percentage was calculated based on individuals who answered having periodic headaches

The mean (SD) number of days with headaches in the past 30 days was 4.6 (5.0), 4.0 (5.0), and 12.5 (10.6) for migraine, tension-type headache, and cluster headache, respectively (Table 1). The mean numbers of days with headaches in the past 3 months were 11.1 (12.3), 9.4 (12.7), and 30.3 (30.3), respectively. The maximum number of headache days in the past 30 days was 30 days for all three types, and 90 or 92 days in the past 3 months. Regarding the age of headache onset, 40.5%, 19.1%, and 57.9% of individuals with migraine, tension-type headache, and cluster headache, respectively reported when they were < 20 years old (Fig. 3A), and 39.9%, 25.8%, and 52.6%, respectively reported that they lived with headaches for ≥ 21 years (Fig. 3B).

**Table 1:** Background characteristics

	**Migraine**		**Tension-type headache**		**Cluster headache**		**Other headache types**	
	**(*n* = 691)**		**(*n* = 1,441)**		**(*n* = 21)**		**(*n* = 5,208)**	
**Variables**	**n**	**%**	**n**	**%**	**n**	**%**	**n**	**%**
Sex								
Male	272	39.4	830	57.6	14	66.7	3,198	61.4
Female	419	60.6	611	42.4	7	33.3	2,010	38.6
Menstruation, yes^a^	325	77.6	454	74.3	6	85.7	1,439	71.6
Age, years								
19–29	60	8.7	115	8.0	2	9.5	284	5.5
30–39	184	26.6	268	18.6	4	19.0	902	17.3
40–49	262	37.9	527	36.6	7	33.3	1,812	34.8
50–59	175	25.3	448	31.1	5	23.8	1,860	35.7
60–74	10	1.4	83	5.8	3	14.3	350	6.7
Member status								
Insured member	582	84.2	1296	89.9	21	100.0	4,714	90.5
Family member	109	15.8	145	10.1	0	0.0	494	9.5
Job category								
Administrative position	231	33.4	400	27.8	9	42.9	1,322	25.4
Professional and technical position	186	26.9	435	30.2	4	19.0	1,381	26.5
Housewife (or husband)	58	8.4	72	5.0	0	0.0	238	4.6
Managers	47	6.8	157	10.9	2	9.5	722	13.9
Other	169	24.5	377	26.2	6	28.6	1,545	29.7
Annual household income^b^ (including tax)								
< 1,000,000 JPY (8,644 USD)	11	1.6	16	1.1	0	0.0	55	1.1
≥ 1,000,000 to < 5,000,000 JPY (8,644 to 43,220 USD)	136	19.7	342	23.7	3	14.3	1,118	21.5
≥ 5,000,000 to < 10,000,000 JPY (43,220 to 86,440 USD)	360	52.1	704	48.9	16	76.2	2,576	49.5
≥ 10,000,000 JPY (86,440 USD)	119	17.2	269	18.7	1	4.8	1,039	20.0
Don't know	54	7.8	93	6.5	1	4.8	327	6.3
No response	11	1.6	17	1.2	0	0.0	93	1.8
Number of days with a headache in the past 30 days								
Mean (SD)	4.6	(5.0)	4.0	(5.0)	12.5	(10.6)	3.3	(4.5)
Median (min, max)	3	(0, 30)	2	(0, 30)	10	(1, 30)	2	(0, 30)
Number of days with a headache in the past 3 months								
Mean (SD)	11.1	(12.3)	9.4	(12.7)	30.3	(30.3)	8.0	(12.0)
Median (min, max)	7	(1, 90)	5	(1, 90)	16	(1, 92)	5	(1, 92)
Receipt code for headache or migraine in the past 6 months^c^								
Yes	61	8.8	38	2.6	5	23.8	144	2.8
Migraine	57	8.2	30	2.1	5	23.8	116	2.2
Tension-type headache	10	1.4	10	0.7	2	9.5	39	0.7
Cluster headache	-	-	-	-	1	4.8	2	0.0
Headache attributed to a vascular disorder or not classified as other	-	-	1	0.1	-	-	-	-
Headache attributed to a substance or not classified as other	-	-	1	0.1	-	-	-	-
Other	-	-	-	-	-	-	1	0.0
Comorbidity in the past 6 months^c^								
Hypertension	51	7.4	171	11.9	3	14.3	633	12.2
Cardiovascular disorders	23	3.3	71	4.9	3	14.3	287	5.5
Cerebrovascular disorders	6	0.9	34	2.4	3	14.3	99	1.9
Gastrointestinal disorders	386	55.9	852	59.1	13	61.9	3,024	58.1
Constipation^d^	50	13.0	81	9.5	3	23.1	299	9.9
Psychiatric and psychosomatic disorders	104	15.1	175	12.1	4	19.0	580	11.1
Depression^d^	48	46.2	92	52.6	1	25.0	287	49.5
Epilepsy	6	0.9	14	1.0	1	4.8	39	0.7
Asthma	51	7.4	84	5.8	0	0.0	274	5.3
Allergy	123	17.8	243	16.9	5	23.8	836	16.1
Autoimmune disorders	38	5.5	78	5.4	1	4.8	224	4.3

*Abbreviations:* *JPY* Japanese yen, *USD* United States dollar, *SD* Standard deviation, *min* Minimum, *max* Maximum

^a^ Denominator was the total number of females

^b^ USD was estimated based on the exchange rate of 1 JPY = 0.008644 USD on 28 January 2022

^c^ Data were derived from the medical claims database. The item “Receipt codes for headache or migraine” was available in the database for individuals who consulted physician and were diagnosed as certain types of headaches

^d^ Denominator for constipation and depression were the total number of individuals with digestive disease and psychiatric and psychosomatic disorders, respectively

### Use of medical care

Overall, 81.0%, 92.0%, and 57.1% of individuals with migraine, tension-type headache, and cluster headache types, respectively, had not seen a doctor (Table 2). The majority of individuals who consulted doctors visited doctors once per month or less. As the reason for initially seeing a doctor, “unable to tolerate headaches” was the most selected response in all three types (15.3%, 7.2%, and 38.1%, respectively), followed by “worried about other brain diseases” in migraine and tension-type headache types (9.4% and 5.9%, respectively) and “increased headache frequency in cluster headache (19.0%). A common reason for seeing a doctor once and not seeing thereafter was “relived not to have a brain disease” (2.6%, 3.3%, and 4.8%, respectively), and “no time” was also selected by 2 individuals with cluster headache. As the reason for not seeing a doctor, “OTC analgesics effective” was commonly selected for migraine (37.6%) and tension-type headache (32.0%) types. The “pain not sufficiently severe” was the most selected response in tension-type headache types (33.4%), whereas “used to having a headache” was the most selected response in cluster headache type (23.8%).

**Table 2:** Use of medical care

	**Migraine**		**Tension-type headache**		**Cluster headache**		**Other headache type**	
	**(*n* = 691)**		**(*n* = 1,441)**		**(*n* = 21)**		**(*n* = 5,208)**	
**Questions and responses**	**n**	**%**	**n**	**%**	**n**	**%**	**n**	**%**
Frequency of current medical visits in the past 6 months								
Regularly visited	44	6.4	37	2.6	4	19.0	124	2.4
Not regularly visited	87	12.6	79	5.5	5	23.8	360	6.9
Not visited	560	81.0	1,325	92.0	12	57.1	4,724	90.7
Frequency of medical visits in the past 6 months								
≥ Once/week	2	0.3	0	0.0	0	0.0	4	0.1
Once/2 weeks	0	0.0	2	0.1	0	0.0	12	0.2
Once/month	22	3.2	14	1.0	2	9.5	40	0.8
Once/2 months	10	1.4	9	0.6	1	4.8	24	0.5
Once/3 months	5	0.7	9	0.6	1	4.8	23	0.4
< Once/3 months	5	0.7	3	0.2	0	0.0	21	0.4
Reasons for initially seeing a doctor for headache (multiple answers)								
Unable to tolerate headaches	106	15.3	104	7.2	8	38.1	355	6.8
Worried about other brain diseases	65	9.4	85	5.9	3	14.3	327	6.3
Increased headache frequency	55	8.0	65	4.5	4	19.0	189	3.6
OTC analgesics no longer effective	48	6.9	29	2.0	3	14.3	107	2.1
Reasons for seeing a doctor once for headache and not seeing thereafter (multiple answers)								
Relieved not to have a brain disease that threatened life	18	2.6	47	3.3	1	4.8	162	3.1
Too much trouble	10	1.4	21	1.5	0	0.0	55	1.1
No time	8	1.2	12	0.8	2	9.5	30	0.6
Prescription drugs ineffective	8	1.2	7	0.5	0	0.0	17	0.3
Reasons for not seeing a doctor in the past 3 years (multiple answers)								
OTC analgesics effective	260	37.6	461	32.0	3	14.3	1,586	30.5
Used to having a headache	155	22.4	186	12.9	5	23.8	545	10.5
Spontaneously resolving after endurance	143	20.7	421	29.2	1	4.8	1,290	24.8
Pain not sufficiently severe	114	16.5	482	33.4	1	4.8	1,734	33.3

*Abbreviation:* *OTC* Over-the-counter

### Clinical features and symptoms

The clinical features and symptoms of each headache type are provided in Table 3. Common symptoms associated with headaches reported in migraine were nausea or vomiting (49.9%), stiff shoulders (35.9%), and neck pain (26.8%); and those reported in tension-type headache were stiff shoulders (14.1%) and neck pain (9.0%). Various symptoms were reported in cluster headache, and the most common symptom was nausea or vomiting (76.2%), followed by stiff shoulder (61.9%), neck pain (61.9%), and teary eye (61.9%). The most common site of pain was unilateral in migraine (84.7%); and additionally periorbital (36.0%) and bilateral (28.7%) were also common sites of pain. Bilateral sites were the most common in tension-type headache (74.7%). In cluster headache, unilateral (81.0%) and periorbital (76.2%) sites were commonly selected, and 23.8% responded “other”.

**Table 3:** Clinical features and symptoms

	**Migraine**		**Tension-type headache**		**Cluster headache**		**Other headache types**	
	**(*n* = 691)**		**(*n* = 1,441)**		**(*n* = 21)**		**(*n* = 5,208)**	
**Questions and responses**	**n**	**%**	**n**	**%**	**n**	**%**	**n**	**%**
Symptoms associated with headache (multiple answers)								
Nausea or vomiting	345	49.9	38	2.6	16	76.2	452	8.7
Stiff shoulder	248	35.9	203	14.1	13	61.9	710	13.6
Neck pain	185	26.8	129	9.0	13	61.9	490	9.4
Photophobia	142	20.5	30	2.1	11	52.4	174	3.3
Phonophobia	118	17.1	30	2.1	7	33.3	128	2.5
Dizziness	101	14.6	58	4.0	8	38.1	217	4.2
Osmophobia	91	13.2	16	1.1	5	23.8	92	1.8
Weakness or lethargy	78	11.3	69	4.8	2	9.5	182	3.5
Teary eye on the side of headache	27	3.9	6	0.4	13	61.9	18	0.3
Site of pain (multiple answers)								
Unilateral	585	84.7	302	21.0	17	81.0	1,662	31.9
Bilateral	198	28.7	1,076	74.7	8	38.1	1,020	19.6
Frontal	181	26.2	323	22.4	8	38.1	1,497	28.7
Occipital	178	25.8	311	21.6	8	38.1	1,383	26.6
Periorbital	249	36.0	319	22.1	16	76.2	1,152	22.1
Other	17	2.5	29	2.0	5	23.8	249	4.8
Time of day (multiple answers)								
Upon waking	195	28.2	315	21.9	7	33.3	1,044	20.0
Morning	143	20.7	311	21.6	5	23.8	823	15.8
Afternoon	302	43.7	516	35.8	7	33.3	1,503	28.9
Evening	142	20.5	229	15.9	6	28.6	704	13.5
Other	13	1.9	11	0.8	0	0.0	70	1.3
No particular time	223	32.3	503	34.9	8	38.1	2,112	40.6
Headache triggers (multiple answers)								
Fatigue	327	47.3	649	45.0	8	38.1	1,966	37.7
Stress	307	44.4	500	34.7	10	47.6	1,679	32.2
Bad weather such as the time of typhoon	286	41.4	374	26.0	7	33.3	1,041	20.0
Lack of sleep	261	37.8	492	34.1	4	19.0	1,495	28.7
Turning points of the seasons	208	30.1	315	21.9	6	28.6	941	18.1
Sunny or rainy days	191	27.6	246	17.1	7	33.3	674	12.9
Work or housework	178	25.8	390	27.1	9	42.9	1,034	19.9
Menstruation	175	25.3	187	13.0	4	19.0	547	10.5
Among females with menstruation^a^	171	52.6	185	40.7	4	66.7	534	37.1
Excessive sleep	165	23.9	194	13.5	4	19.0	606	11.6
Feeling nervous	149	21.6	207	14.4	6	28.6	575	11.0
Weekdays	120	17.4	215	14.9	7	33.3	592	11.4
Weekend (including holidays)	111	16.1	130	9.0	7	33.3	302	5.8
Drinking alcohol	105	15.2	132	9.2	6	28.6	424	8.1
Release from nervousness	77	11.1	39	2.7	3	14.3	192	3.7
Release from stress	69	10.0	32	2.2	1	4.8	149	2.9
No particular triggers	60	8.7	183	12.7	2	9.5	934	17.9
Smell of perfume or cigarettes	53	7.7	51	3.5	4	19.0	153	2.9
Sleep	48	6.9	43	3.0	6	28.6	136	2.6
Activities interfered by headache (multiple answers)								
No focus on work or study	480	69.5	801	55.6	16	76.2	2,686	51.6
Unable or unwilling to conduct housework	367	53.1	396	27.5	9	42.9	1,246	23.9
Unwilling to work or study	297	43.0	515	35.7	13	61.9	1,464	28.1
Cancelling plans or appointments	197	28.5	129	9.0	4	19.0	429	8.2
Absence from work or school	189	27.4	142	9.9	5	23.8	506	9.7
Unable to go outside	179	25.9	108	7.5	7	33.3	387	7.4
Unable to stay in crowded places	166	24.0	108	7.5	5	23.8	351	6.7

^a^Denominator was the total number of females with menstruation

Regardless of headache type, the common time of day of headache onset was afternoon (43.7%, 35.8%, and 33.3% for migraine, tension-type headache, and cluster headache, respectively) and “no particular time of day” (32.3%, 34.9%, and 38.1%, respectively) (Table 3). When stratified by age, the general pattern did not change, but in ages ≤ 29 years old, time of day was evenly selected with relatively large proportion of headache onset in the evening (Additional file 2). Upon waking and morning were commonly selected by people 60*–*74 years old in migraine and tension-type headache.

Regarding headache triggers, fatigue and stress were most commonly selected in migraine (47.3% and 44.4%, respectively) and tension-type headache (45.0% and 34.7%, respectively) (Table 3). Weather-related triggers and turning of the seasons (bad weather such as the time of typhoon, 41.4%; turning points of the seasons, 30.1%; sunny or rainy days, 27.6%) were also commonly selected for migraine. In cluster headache, in addition to the above responses, drinking alcohol (28.6%) and sleep (28.6%) were also commonly selected. In all three types among women with menstruation, menstruation was a common trigger (52.6%, 40.7%, and 66.7% in migraine, tension-type headache, and cluster headache, respectively).

Headaches interfered with focus on work or study in a large proportion of individuals in all three types (69.5%, 55.6%, and 76.2% in migraine, tension-type headache, and cluster headache types, respectively), and “unable or unwilling to housework” and “unwilling to work or study” were also commonly selected in migraine (53.1% and 43.0%, respectively) and cluster headache (42.9% and 61.9%, respectively) (Table 3). Headaches refrained or reduced a wide range of activities, and “operating a computer or smartphone” and “drinking alcohol” were commonly selected by both men and women in all three types (Table 4). In migraine and tension-type headaches, more women than men responded that headaches refrained or reduced housework.

**Table 4:** Activities refrained from or reduced by headaches

	**Migraine (*n* = 691)**				**Tension-type headache (*n* = 1,441)**				**Cluster headache (*n* = 21)**				**Other headache types (*n* = 5,208)**			
	**Male (*n* = 272)**		**Female (*n* = 419)**		**Male (*n* = 830)**		**Female (*n* = 611)**		**Male (*n* = 14)**		**Female (*n* = 7)**		**Male (*n* = 3,198)**		**Female (*n* = 2,010)**	
**Responses (multiple answers)**	**n**	**%**	**n**	**%**	**n**	**%**	**n**	**%**	**n**	**%**	**n**	**%**	**n**	**%**	**n**	**%**
Operating a computer or smartphone	89	32.7	147	35.1	231	27.8	162	26.5	5	35.7	3	42.9	807	25.2	513	25.5
Drinking alcohol	88	32.4	126	30.1	215	25.9	157	25.7	8	57.1	2	28.6	778	24.3	454	22.6
Going to crowded places	58	21.3	157	37.5*	122	14.7	165	27.0*	2	14.3	3	42.9	368	11.5	467	23.2*
Exercising such as playing sports or walking	58	21.3	71	16.9	99	11.9	65	10.6	3	21.4	2	28.6	380	11.9	225	11.2
Driving a car	45	16.5	47	11.2*	83	10.0	51	8.3	3	21.4	2	28.6	300	9.4	158	7.9
Housework (excluding grocery shopping, laundry, and cooking)	31	11.4	91	21.7*	44	5.3	132	21.6*	2	14.3	0	0.0	174	5.4	357	17.8*
Socializing with friends and playing with children	25	9.2	58	13.8	40	4.8	52	8.5*	1	7.1	1	14.3	111	3.5	162	8.1*
Going to grocery shopping	18	6.6	90	21.5*	32	3.9	101	16.5*	1	7.1	2	28.6	111	3.5	308	15.3*
Taking public transportation	14	5.1	51	12.2*	17	2.0	36	5.9*	0	0.0	2	28.6*	93	2.9	102	5.1*
Cooking	11	4.0	93	22.2*	16	1.9	104	17.0*	1	7.1	1	14.3	82	2.6	300	14.9*
Taking a bath	11	4.0	35	8.4*	18	2.2	26	4.3*	2	14.3	0	0.0	77	2.4	94	4.7*
Doing laundry	8	2.9	45	10.7*	9	1.1	42	6.9*	0	0.0	2	28.6*	43	1.3	111	5.5*
Dropping-off and picking-up children or family members	6	2.2	20	4.8	5	0.6	8	1.3	0	0.0	2	28.6*	26	0.8	30	1.5*
Socializing with neighbors	4	1.5	32	7.6*	13	1.6	16	2.6	0	0.0	1	14.3	54	1.7	61	3.0*
Putting on make-up	0	0.0	25	6.0*	1	0.1	18	2.9*	0	0.0	0	0.0	1	0.0	63	3.1*
Other	82	30.1	87	20.8*	294	35.4	159	26.0*	3	21.4	2	28.6	1,236	38.6	537	26.7*

The Chi-square test was used to compare percentages between sexes for each response, with a two-sided significance level of *p* < 0.05 presented with an asterisk (*)

### Medication use

OTC analgesics were commonly used in all three types (89.6%, 88.4%, and 85.0% in migraine, tension-type headache, and cluster headache, respectively), and 22.9%, 13.0%, and 28.6%, respectively used ≥ 2 OTC analgesics (Table 5). Prophylactic drug use was uncommon in all three types, and among acute drugs, acetaminophen and NSAIDs were commonly used (29.8%, 28.4%, and 42.9%, respectively). Triptan was used in 5.9% of individuals with migraine.

**Table 5:** Medication use

	**Migraine**		**Tension-type headache**		**Cluster headache**		**Other headache types**	
	**(*n* = 691)**		**(*n* = 1,441)**		**(*n* = 21)**		**(*n* = 5,208)**	
**Variables**	**n**	**%**	**n**	**%**	**n**	**%**	**n**	**%**
Medication use in the past 6 months, yes	626	90.6	978	67.9	20	95.2	3,411	65.5
No prescription drugs^a^	27	4.3	64	6.5	3	15.0	264	7.7
OTC analgesics only^a^	362	57.8	559	57.2	6	30.0	1,959	57.4
Prescription drugs only (acute and prophylactic)^a^	38	6.1	49	5.0	0	0.0	161	4.7
OTC and prescription drugs^a^	199	31.8	306	31.3	11	55.0	1,027	30.1
Number of OTC analgesic types								
1	403	58.3	677	47.0	11	52.4	2,420	46.5
≥ 2	158	22.9	188	13.0	6	28.6	566	10.9
Types of prescription drugs in the past 6 months (prophylactic)								
Antidepressants	23	3.3	45	3.1	0	0.0	137	2.6
Anti-epileptics	13	1.9	21	1.5	1	4.8	78	1.5
Calcium channel blockers	11	1.6	21	1.5	2	9.5	57	1.1
ARB/ACE inhibitors	6	0.9	27	1.9	0	0.0	112	2.2
Beta-blockers	4	0.6	8	0.6	0	0.0	21	0.4
Other	6	0.9	17	1.2	1	4.8	55	1.1
Types of prescription drugs in the past 6 months (acute)								
Acetaminophen/NSAIDs	206	29.8	409	28.4	9	42.9	1,435	27.6
Triptans	41	5.9	13	0.9	4	19.0	59	1.1
Antiemetics	28	4.1	25	1.7	1	4.8	118	2.3
Intravenous steroids	14	2.0	23	1.6	0	0.0	91	1.7
Tranquilizers/anesthetic preparations	6	0.9	12	0.8	0	0.0	62	1.2
Tramadol	1	0.1	2	0.1	0	0.0	9	0.2
Magnesium preparations	1	0.1	0	0.0	0	0.0	4	0.1
Ergotamine	0	0.0	0	0.0	0	0.0	0	0.0

Use of OTC analgesics, no prescription drug use, and number of OTC analgesic types were retrieved from the questionnaire responses, and prescription drugs data were derived from the medical claims database

*Abbreviations:* *OTC* Over-the-counter, *ARB* Angiotensin-receptor blocker, *ACE* Angiotensin converting enzyme inhibitor, *NSAIDs* Non-steroidal anti-inflammatory drugs

^a^ Denominator was the number of individuals who answered "yes" to a question of taking any medications

### Severity of pain and activity impairment

Among individuals taking medicines, 16.8% and 15.8% in the migraine and tension-type headache groups reported moderate to severe pain, respectively, whereas approximately half of the individuals with cluster headache reported these levels of pain (47.6%) (Table 6). Approximately over two thirds of individuals with migraine and cluster headache reported moderate or severe impairment in daily activities when not taking medicines (72.9% and 85.7%, respectively), and 12.6% and 19.0% of those respectively reported these levels of impairment when taking medicines. The percentage difference between individuals with tension-type headache not taking and taking medicines was relatively small, but the percentage was smaller in those taking medicines (27.3% and 7.7%, respectively). For the question “hoped reduction in headache”, “almost none” was most commonly selected in all three types (54.8%, 42.8%, and 61.9%, respectively).

**Table 6:** Severity of pain and activity impairment

	**Migraine (*n* = 691)**				**Tension-type headache (*n* = 1,441)**				**Cluster headache (*n* = 21)**				**Other headache types (*n* = 5,208)**			
	**When not taking medicines (*n* = 691)**		**When taking medicines (*n* = 626)**		**When not taking medicines (*n* = 1,441)**		**When taking medicines (*n* = 978)**		**When not taking medicines (*n* = 21)**		**When taking medicines (*n* = 20)**		**When not taking medicines (*n* = 5,208)**		**When taking medicines (*n* = 3,411)**	
**Variables**	**n**	**%**	**n**	**%**	**n**	**%**	**n**	**%**	**n**	**%**	**n**	**%**	**n**	**%**	**n**	**%**
Severity of pain^a^ (single answer)																
Severe	365	52.8	47	6.8	119	8.3	89	6.2	21	100.0	4	19.0	1,069	20.5	243	4.7
Moderate	316	45.7	69	10.0	841	58.4	138	9.6	0	0.0	6	28.6	2,112	40.6	514	9.9
Mild	10	1.4	510	73.8	481	33.4	751	52.1	0	0.0	10	47.6	2,027	38.9	2,654	51.0
Impairment in daily activities^b^ (single answer)																
Severe	204	29.5	31	4.5	62	4.3	26	1.8	13	61.9	1	4.8	395	7.6	76	1.5
Moderate	300	43.4	56	8.1	332	23.0	85	5.9	5	23.8	3	14.3	1,218	23.4	263	5.0
Mild	187	27.1	539	78.0	1,047	72.7	867	60.2	3	14.3	16	76.2	3,595	69.0	3,072	59.0
Hoped reduction in the number of headache in a month for improving daily life (single answer)																
Slight	201		29.1		405		28.1		1		4.8		1,433		27.5	
Almost half	30		4.3		65		4.5		2		9.5		208		4.0	
Almost none	379		54.8		617		42.8		13		61.9		2,046		39.3	
Reduction in pain intensity per attack rather than reduction in pain frequency	70		10.1		87		6.0		4		19.0		395		7.6	

^a^ Severity of pain in 5-point scale was categorized as following: severe = extreme pain or quite a bit of pain, moderate = moderate pain, and mild = little pain or no pain

^b^ Impairment in daily activities in 5-point scale was categorized as following: severe = extreme difficulty or severe disruption in daily life, moderate = moderate difficulty in daily life, and mild = slightly interferes with daily life or no trouble at all

**Fig. 4:**
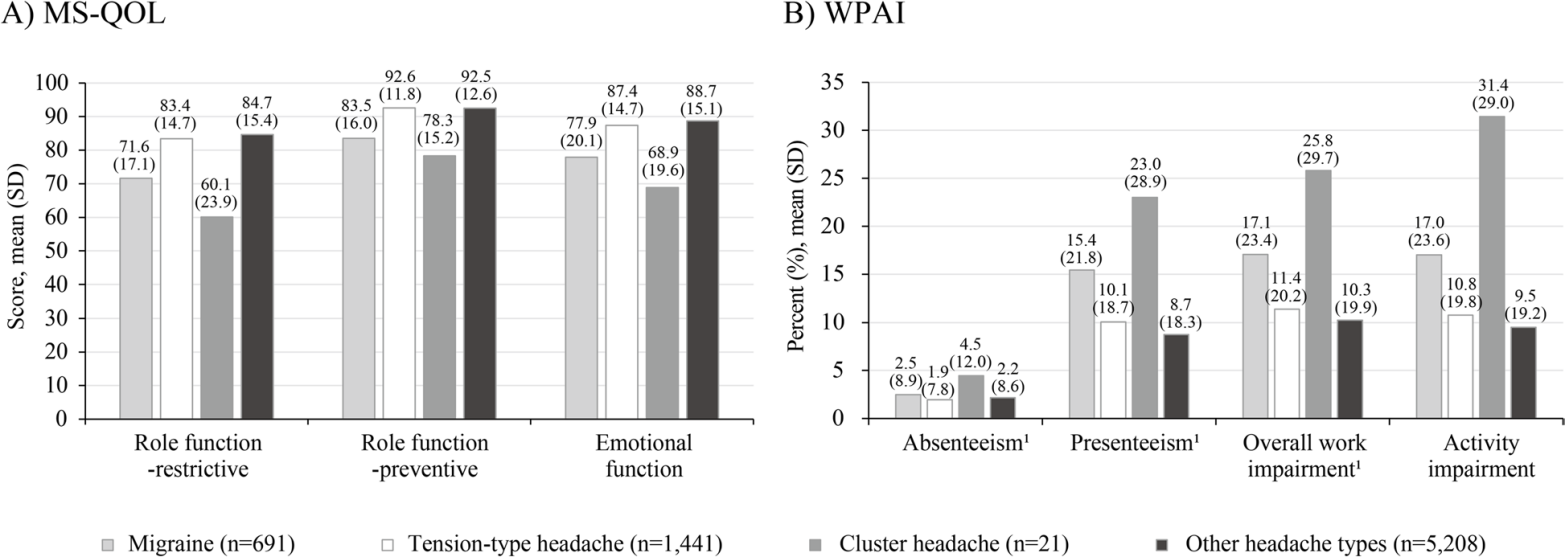
MS-QOL (**A**) and WPAI (**B**). Abbreviations: MS-QOL; migraine-specific quality of life; WPAI, Work Productivity Activity Impairment; SD, standard deviation. Notes: ^1^The number of individuals with responses examined were 605, 1293, 20, and 4627 individuals with migraine, tension-type headache, cluster headache, and other headaches, respectively for all variables, except activity impairment

The mean (SD) MS-QOL scores and percentages of WPAI scores are provided in Fig. 4. Although not statistically tested due to some patients being included in two primary headache groups, irrespective of headache types, absenteeism was the lowest among the four WPAI components. The percentage of all components except absenteeism was lower in tension-type headache than in migraine.

## Discussion

This study reported the up-to-date epidemiological data and impact of primary headaches on daily activities among people with primary headaches classified according to the ICHD-3 using a nationwide online survey coupled with medical claims. Our study also revealed wealth of information collected from nationwide individuals on clinical features and burden of several headache types including tension-type headache for which the information was previously largely missing. This is because, tension-type headaches do not greatly interfere with daily activities, and therefore presumably only few people visit medical institutions. For cluster headache, our study is one of the first to report its epidemiological data from nationwide survey.

The prevalence of migraine and tension-type headache was 4.3 and 2.0 times higher in women than in men, respectively, and was higher in people in their 20 s to 40 s, the findings generally consistent with previous reports in Japan [5, 6]. Although the direct comparison is not possible, a higher prevalence of migraine in women than in men was also found in a study in Korea [20] and in Japan [11]. In contrast to our findings, these studies reported a lower prevalence of tension-type headache in women [11, 20]. One of the possible reasons for such differences may be headaches being more common in these study populations of workers from IT companies. The former study found that one-fifth of the populations had experienced migraine, and the number increased to one-third for tension-type headache [20]. Additionally, approximately over half of the workers experienced work-related triggers of headache (e.g., overtime, prolonged computer use, and heavy workload), and different tendencies may be partly attributable to background differences in our study and these studies. The prevalence of cluster headaches appeared to be higher in men in their 20 s. The numbers of individuals in each age category were however small, and more data are needed to clarify this result. We found 39.9% and 52.6% in migraine and cluster headache types, respectively lived with headache for ≥ 21 years. Migraine, as aforementioned, and cluster headaches are prevalent in young ages [21] and can have a long course of illness. Our findings are therefore consistent with those of previous reports. As our result is based on individuals who answered having periodic headaches and there were individuals who did not remember the age of headache onset, the result needs to be carefully interpreted.

Approximately 57%–92% of people with three headache types did not see doctors, and medical consultation rates were low in all three headache types, which was in line with previous reports in people with migraine [5, 6]. OTC analgesics were commonly used in all three headache types (approximately 85%–90% depending on headache types), and relatively a large proportion of people responded that they do not see doctors because OTC analgesics are effective (approximately 14%–38%). However, the most common reason for seeing a doctor was "unable to tolerate headache" in all headache types, and the response “OTC analgesics no longer effective” was also commonly selected. Although each of these cases need to be examined closely, common use of and perceived ineffectiveness of OTC analgesics may be related to lack of insight on headaches and medications. OTC analgesics are widely promoted via media, and this promotion may have influence on widespread OTC analgesics use and low consultation rates. Lack of understanding by people around them at workplace or social environment [22] may also be one possible reason of low consultation rates. The most common reason for not seeing a doctor in cluster headache was "used to having a headache". Untreated attacks of cluster headaches last for 15–180 min [23], and this duration is relatively short compared to that of other headache types. As the symptoms can resolve after a certain period of time (cluster period), some people presumably misunderstood that OTC analgesics effectively ameliorated pain. These findings underscore the need for further education on disease and medications.

Although relatively uncommon, secondary headaches with underlying causes may be present in some individuals. A previous study in Japan found that 27 out of 334 patients (8.1%) with headaches requiring emergency care had subarachnoid hemorrhage (SAH) [24]. Furthermore, another study in the United States reported that 12% (56 of 482) of patients diagnosed as SAH were initially misdiagnosed, and among the misdiagnosed patients, the most common misdiagnosis was migraine or tension-type headache (36%) [25]. Although life-threatening cases may be relatively uncommon, it is advisable for individuals experiencing headaches to seek medical care.

We documented some common symptoms and pain sites across headache types including stiff shoulders and bilateral pain in migraine and tension-type headache, the latter reported in Takeshima et al. [6], and unilateral and periorbital pain in migraine and cluster headache. These overlapping symptoms and pain sites suggest that it is essential to carefully interview patients for symptoms during diagnosis. These findings may also suggest that it may not be possible to diagnose headache types based on questionnaire alone, as discussed in Sakai et al. [13] for migraine.

Afternoon was the common time of day of headache onset in all three headache types. This result differed from previous reports of migraine in which migraine attacks tended to occur in the morning; however, some recent studies also reported migraine attacks in the afternoon and evening [26]. We speculate that one of the reasons for these differences may be the changes in the environment. Computers, smartphones, and tablets are now routinely used in our work and educational environment, and headache attacks may occur when fatigue is accumulated and physical and mental stress are released after work or school in the afternoon. People in their 20 s also commonly experience headache attacks in the evening. This finding may be related to the use of electronic gadgets by this generation in the evening. Headaches in people in their 50 s and 60 s were also common upon waking and in the morning, and it is possible that some of these headaches are morning headache which is related to other disease including sleep apnoea [27] and may require medical examination.

The most commonly reported headache trigger was fatigue for both migraine and tension-type headache, and stress, work or housework, and lack of sleep were also common triggers, as previously reported in people with migraine and tension-type headache [5, 6]. Additionally, the results showed that weather-related phenomena and seasonal changes were common triggers among people with migraine. Individuals with cluster headaches reported that headaches occurred while sleeping or drinking, which is consistent with our clinical observations. Menstruation-related headaches were also common in all headache types (approximately 41%–67% of individuals with menstruation depending on headache types). This result was in line with the observation in clinical practice where we observed that about half of the women with these headaches had menstruation-related migraine headaches.

The top three activities refrained from or reduced by headaches were “operating a computer or smartphone”, “drinking alcohol”, and “going to crowded places” in all three headache types. As the prevalence of these headaches was high among individuals in their 20 s to 40 s, which is the prime age of work and social activities, our findings suggest that activities that are often difficult to avoid in working and social environments may be refrained from or reduced by headaches. Furthermore, women with migraine and tension-type headache commonly responded that housework-related activities were refrained from or reduced by their headaches. In clinical practice, we observed that some female patients felt sorry for not being able to conduct housework. While women’s participation in the society has been increasing in Japan, men’s cooperation in housework has not yet become widespread.

As a reference, we reported the mean MS-QOL scores among the three headache types, and the scores appeared to be higher in tension-type headache and lower in cluster headache than in migraine. The score difference between tension-type headache and migraine was smaller than expected, and this result suggest that more severe tension-type headache such as transformed migraine [28] might be included in this group. Additionally, larger proportions of people with migraine experienced severe or moderate disability in daily activities than people with tension-type headache regardless of medication status. Although the outcome measure differed from our study, similar tendency was reported in a previous study, which showed greater disability in terms of migraine disability assessment scores in migraine than tension-type headache [20]. WPAI scores for migraine in terms of presenteeism, overall work impairment, and activity impairment were lower in our study than those in recent studies in people with migraine (approximately 30%–55%) [7, 9]. As the majority of our study population was active workers, it is possible that the distribution of pain and impairment may be leaned towards mild levels. Nonetheless some treated individuals in our study reported moderate or severe pain (approximately 16%–48% depending on headache types) and impairment in daily activities (approximately 8%–19%). These people therefore may require medical intervention to ameliorate pain and improve their quality of life, and relatively low MS-QOL scores and high WPAI scores of cluster headache, although statistical comparison was not made among headache types in our study, warrant further investigation.

### Limitations

This study has limitations similar to those described by Sakai et al. [13]. Our findings are not generalizable to the entire adult population with headache in Japan for the following reasons. First, we used data of employees and their family members in large companies that are members of the health insurance association contracted by DeSC. Self-employed persons, civil servants, employees of small- and medium-sized companies, and retired elderly persons were not included. Second, the questionnaire was distributed only to the kencom^®^ users. The users are considered more health-conscious than non-users and are more likely to take positive health actions in their daily lives, which might have affected their QOL and WPAI scores. Third, headache types were classified according to the ICHD-3 based on online surveys, and detailed information regarding symptoms was not available in the database. As described by Sakai et al. [13], the “other headache types” may include individuals with migraine. Lastly, a large portion of our data was self-reported, and questionnaire responses are subject to recall bias. Such bias is not present in certain variables (e.g., drug prescriptions and comorbidities); however, as we used an existing medical claims database that is used for billing purposes, the data are also subject to misclassification and entry error. The MS-QOL scores for people with non-migraine headache types were not intended for direct comparison.

## Conclusions

Based on a novel method of linking medical claims and online surveys, this study reported the up-to-date epidemiological data of several headache types and their impact on daily activities in Japan and identified new findings including various triggers of headache attacks and daily activities refrained from or reduced by the headaches. Furthermore, this study, for the first time, suggested the disease burden in people possibly experiencing tension-type headaches, many of whom had not yet seen a doctor. Our study suggests that although not as much as migraine, tension-type headaches may negatively impact quality of life. We consider that the findings of this study are of clinical value for the diagnosis and treatment of primary headaches.

## Supplementary Information

**Additional file 1.** Classification of migraine, tension-type headache, and cluster headache.

**Additional file 2.** Time of day of headache onset.

## Data Availability

The data that support the findings of this study are available from DeSC Healthcare, Inc. (Tokyo, Japan) but restrictions apply to the availability of these data, which were used under license for the current study, and so are not publicly available. Data are however available from the authors (HS) upon reasonable request and with permission of DeSC Healthcare, Inc.

## Abbreviations

ACE: Angiotensin converting enzyme inhibitor
ARB: Angiotensin-receptor blocker
ICHD-3: International classification of headache disorders, 3rd edition
JPY: Japanese yen
max: Maximum
min: Minimum
MS-QOL: Migraine-specific quality of life
NSAIDs: Non-steroidal anti-inflammatory drugs
OTC: Over-the-counter
SD: Standard deviation
USD: United States dollar
WPAI: Work productivity activity impairment
