# Supplementary material for: Clinical characteristics, medication use, and impact of primary headache on daily activities: an observational study using linked online survey and medical claims data in Japan
Source: BMC Neurol. 2023 Feb 21;23:80. doi: 10.1186/s12883-023-03122-9 (PMC9942338; doi:10.1186/s12883-023-03122-9)
Supplement: Supplementary file 1 — Additional file 1. Classification of migraine, tension-type headache, and cluster headache. [file 12883_2023_3122_MOESM1_ESM.docx]

# **Additional files**

## **Additional file 1** Classification of migraine, tension-type headache, and cluster headache

|  | **Migraine** | | | **Tension-type headache** | | | **Cluster headache** | | | |
| --- | --- | --- | --- | --- | --- | --- | --- | --- | --- | --- |
| Headache lasting for 7 days to 1 year (single answer) | - | | | - | | | a) is selected | | | |
| a) Yes | - | | | - | | | 〇 | | | |
| b) No | - | | | - | | |  | | | |
| Duration (single answer) | At least b), c), or d) is selected | | | Any of the responses is selected | | | a) is selected | | | |
| a) <4 hours |  | | | 〇 | | | 〇 | | | |
| b) Half a day | 〇 | | |  |  |  |  | | | |
| c) All day |  |  |  |  |  |  |  | | | |
| d) 2 to 3 days |  |  |  |  |  |  |  | | | |
| e) 4 to 14 days |  | | |  |  |  |  | | | |
| f) ≥15 days |  | | |  |  |  |  | | | |
| Site of pain (multiple answers) | At least a) is selected | | | At least b) is selected | | | a) and c) | a) and e) | a), c), and e) | |
| a) Unilateral | 〇 | | |  | | | 〇 | 〇 | 〇 | |
| b) Bilateral |  | | | 〇 | | |  |  |  | |
| c) Frontal |  | | |  | | | 〇 |  | 〇 | |
| d) Occipital |  | | |  | | |  |  |  | |
| e) Periorbital |  | | |  | | |  | 〇 | 〇 | |
| f) Other |  | | |  | | |  |  |  | |
| Characteristics (multiple answers) | At least a) or g) is selected | | | At least b) is selected | | | - | | | |
| a) Throbbing or pulsating pain | 〇 | | |  | | | - | | | |
| b) Tightening pain |  | | | 〇 | | | - | | | |
| c) Prickling pain |  | | |  | | | - | | | |
| d) Tingling pain |  | | |  | | | - | | | |
| e) Gouged pain behind the eye |  | | |  | | | - | | | |
| f) Burning pain |  | | |  | | | - | | | |
| g) Pounding pain^2^ | 〇 | | |  | | | - | | | |
| h) Cracking pain (like being hit by a hammer) |  | | |  | | | - | | | |
| i) Heavy-headed |  | | |  | | | - | | | |
| j) Other |  | | |  | | | - | | | |
| Change in severity due to daily activities (walking, climbing up-stairs, etc.) or due to physical activity (single answer) | At least a) in “Change in severity due to daily activities” or a) in “State when in pain” is selected | | | At least b) or c) in “Change in severity due to daily activities” or at least b) in “State when in pain” is selected | | | - | | | |
| a) Worsens (avoid movement due to pain) | 〇 | | |  | | | - | | | |
| b) No change |  | | | 〇 | | | - | | | |
| c) Gets better |  | | |  |  |  | - | | | |
| d) Sometimes gets better and sometimes gets worse |  | | |  | | | - | | | |
| e) I don't know |  | | |  | | | - | | | |
| State when in pain (single answer) | At least a) in “Change in severity due to daily activities” or a) in “State when in pain” is selected | | | At least b) or c) in “Change in severity due to daily activities” or at least b) in “State when in pain” is selected | | | At least c) in “State when in pain” or at least e), f), or g) in “Symptom associated with headache” is selected | | | |
| a) It is more comfortable to stay still | 〇 | | |  | | |  | | | |
| b) Staying still does not change the severity of pain |  | | | 〇 | | |  | | | |
| c) Pain makes it hard to stay still |  | | |  | | | 〇 | | | |
| d) I don't know |  | | |  | | |  | | | |
| Symptom associated with headache (multiple answers) | a) | b) and c) | a), b), and c) | At least one response from h) to k) but a) to c) are not selected | b) and one response from h) to k) | c) and one response from h) to k) | At least c) in “State when in pain” or at least e), f), or g) in “Symptom associated with headache” is selected | | |  |
| a) Nausea or vomiting | 〇 |  | 〇 | × |  |  |  | | |  |
| b) Photophobia |  | 〇 | 〇 |  | 〇 |  |  | | |  |
| c) Phonophobia |  | 〇 | 〇 |  |  | 〇 |  | | |  |
| d) Osmophobia |  |  |  |  |  |  |  | | |  |
| e) Bloodshot eye on the side of headache |  |  |  |  |  |  | 〇 | | |  |
| f) Teary eye on the side of headache |  |  |  |  |  |  |  |  |  |  |
| g) Runny nose on the side of headache |  |  |  |  |  |  |  |  |  |  |
| h) Dizziness |  |  |  | 〇 | 〇 | 〇 |  | | |  |
| i) Weakness or lethargy |  |  |  |  |  |  |  | | |  |
| j) Stiff shoulders |  |  |  |  |  |  |  | | |  |
| k) Stiff neck |  |  |  |  |  |  |  | | |  |
| l) Numbness in hands and feet |  |  |  |  |  |  |  | | |  |
| m) Other |  |  |  |  |  |  |  | | |  |
| Severity (when not taking medicines) (single answer) | At least one of c), d), or e) is selected | | | At least b) or c) is selected | | | At least d) or e) is selected | | | |
| a) No pain |  | | |  | | |  | | | |
| b) Little pain |  | | | 〇 | | |  | | | |
| c) Moderate pain | 〇 | | |  |  |  |  | | | |
| d) Quite a bit of pain |  |  |  |  | | | 〇 | | | |
| e) Extreme pain |  |  |  |  | | |  |  |  |  |
| **Additional information** | If only one of the six criteria above did not apply, the patient was considered to have a "probable migraine" and was included in the migraine category. | | | If only one of the six criteria above did not apply, the patient was considered to have a "probable tension-type headache" and was included in the tension-type headache category. | | | If only one of the six criteria above did not apply, the patient was considered to have a "probable cluster headache" and was included in the cluster headache category. | | | |

**Notes:** This study used the classification used in Sakai et al. [1].

**Reference**

1. Sakai F, Hirata K, Igarashi H, Takeshima T, Nakayama T, Sano H, et al. (2022) A study to investigate the prevalence of headache disorders and migraine among people registered in a health insurance association in Japan. J Headache Pain 23(1):70. doi: 10.1186/s10194-022-01439-3.
