# Supplementary material for: Clinical characteristics, medication use, and impact of primary headache on daily activities: an observational study using linked online survey and medical claims data in Japan
Source: BMC Neurol. 2023 Feb 21;23:80. doi: 10.1186/s12883-023-03122-9 (PMC9942338; doi:10.1186/s12883-023-03122-9)
Supplement: Supplementary file 2 — Additional file 2. Time of day of headache onset. [file 12883_2023_3122_MOESM2_ESM.docx]

## **Additional file 2** Time of day of headache onset

|  | **Migraine** | | **Tension-type headache** | | **Cluster headache** | | **Other headache types** | |
| --- | --- | --- | --- | --- | --- | --- | --- | --- |
|  | **(n=691)** | | **(n=1,441)** | | **(n=21)** | | **(n=5,208)** | |
| **Age, Time of day (multiple answers)** | **n** | **%** | **n** | **%** | **n** | **%** | **n** | **%** |
| 19-29 years old | 60 |  | 115 |  | 2 |  | 284 |  |
| Upon waking | 16 | 26.7 | 16 | 13.9 | 1 | 50.0 | 41 | 14.4 |
| Morning | 15 | 25.0 | 19 | 16.5 | 1 | 50.0 | 39 | 13.7 |
| Afternoon | 33 | 55.0 | 52 | 45.2 | 0 | 0.0 | 105 | 37.0 |
| Evening | 16 | 26.7 | 25 | 21.7 | 0 | 0.0 | 50 | 17.6 |
| Other | 0 | 0.0 | 0 | 0.0 | 0 | 0.0 | 0 | 0.0 |
| No particular time | 14 | 23.3 | 39 | 33.9 | 1 | 50.0 | 109 | 38.4 |
| 30-39 years old | 184 |  | 268 |  | 4 |  | 902 |  |
| Upon waking | 52 | 28.3 | 61 | 22.8 | 0 | 0.0 | 142 | 15.7 |
| Morning | 34 | 18.5 | 59 | 22.0 | 0 | 0.0 | 119 | 13.2 |
| Afternoon | 90 | 48.9 | 105 | 39.2 | 3 | 75.0 | 332 | 36.8 |
| Evening | 44 | 23.9 | 45 | 16.8 | 0 | 0.0 | 162 | 18.0 |
| Other | 3 | 1.6 | 3 | 1.1 | 0 | 0.0 | 10 | 1.1 |
| No particular time | 51 | 27.7 | 97 | 36.2 | 1 | 25.0 | 367 | 40.7 |
| 40-49 years old | 262 |  | 527 |  | 7 |  | 1,812 |  |
| Upon waking | 67 | 25.6 | 104 | 19.7 | 2 | 28.6 | 369 | 20.4 |
| Morning | 52 | 19.8 | 126 | 23.9 | 2 | 28.6 | 299 | 16.5 |
| Afternoon | 107 | 40.8 | 197 | 37.4 | 2 | 28.6 | 540 | 29.8 |
| Evening | 49 | 18.7 | 98 | 18.6 | 3 | 42.9 | 235 | 13.0 |
| Other | 3 | 1.1 | 5 | 0.9 | 0 | 0.0 | 23 | 1.3 |
| No particular time | 100 | 38.2 | 166 | 31.5 | 3 | 42.9 | 750 | 41.4 |
| 50-59 years old | 175 |  | 448 |  | 5 |  | 1,860 |  |
| Upon waking | 58 | 33.1 | 110 | 24.6 | 4 | 80.0 | 421 | 22.6 |
| Morning | 38 | 21.7 | 98 | 21.9 | 1 | 20.0 | 306 | 16.5 |
| Afternoon | 69 | 39.4 | 140 | 31.3 | 1 | 20.0 | 462 | 24.8 |
| Evening | 31 | 17.7 | 52 | 11.6 | 2 | 40.0 | 212 | 11.4 |
| Other | 6 | 3.4 | 3 | 0.7 | 0 | 0.0 | 30 | 1.6 |
| No particular time | 55 | 31.4 | 168 | 37.5 | 1 | 20.0 | 744 | 40.0 |
| 60-74 years old | 10 |  | 83 |  | 3 |  | 350 |  |
| Upon waking | 2 | 20.0 | 24 | 28.9 | 0 | 0.0 | 71 | 20.3 |
| Morning | 4 | 40.0 | 9 | 10.8 | 1 | 33.3 | 60 | 17.1 |
| Afternoon | 3 | 30.0 | 22 | 26.5 | 1 | 33.3 | 64 | 18.3 |
| Evening | 2 | 20.0 | 9 | 10.8 | 1 | 33.3 | 45 | 12.9 |
| Other | 1 | 10.0 | 0 | 0.0 | 0 | 0.0 | 7 | 2.0 |
| No particular time | 3 | 30.0 | 33 | 39.8 | 2 | 66.7 | 142 | 40.6 |
